# The Influence of Some Axial Ligands on Ruthenium–Phthalocyanine Complexes: Chemical, Photochemical, and Photobiological Properties

**DOI:** 10.3389/fmolb.2020.595830

**Published:** 2021-01-12

**Authors:** Tássia Joi Martins, Laisa Bonafim Negri, Laena Pernomian, Kelson do Carmo Freitas Faial, Congcong Xue, Regina N. Akhimie, Michael R. Hamblin, Claudia Turro, Roberto S. da Silva

**Affiliations:** ^1^Department of Chemistry, Faculty of Philosophy, Sciences and Letters of Ribeirão Preto University of São Paulo, Ribeirão Preto, Brazil; ^2^Department of Chemistry and Biochemistry, The Ohio State University, Columbus, OH, United States; ^3^Department of Physics and Chemistry, School of Pharmaceutical Sciences of Ribeirão Preto, University of São Paulo, Ribeirão Preto, Brazil; ^4^Wellman Center for Photomedicine, Massachusetts General Hospital, Boston, MA, United States; ^5^Department of Dermatology, Harvard Medical School, Boston, MA, United States; ^6^Department of Pharmacology of the School of Medicine of Ribeirão Preto, University of São Paulo, Ribeirão Preto, Brazil; ^7^Environmental Section, Evandro Chagas Institute (SAMAM/IEC), Ananindeua, Brazil; ^8^Laser Research Center, Faculty of Health Sciences, University of Johannesburg, Johannesburg, South Africa

**Keywords:** photodynamic therapy, photobiological assays, ruthenium-phthalocyanine complexes, B16F10 murine melanoma cells, cell viability

## Abstract

This work presents a new procedure to synthesize ruthenium–phthalocyanine complexes and uses diverse spectroscopic techniques to characterize *trans-*[RuCl(Pc)DMSO] **(I)** (Pc = phthalocyanine) and *trans*-[Ru(Pc)(4-ampy)_2_] **(II)** (4-ampy = 4-aminopyridine). The triplet excited-state lifetimes of **(I)** measured by nanosecond transient absorption showed that two processes occurred, one around 15 ns and the other around 3.8 μs. Axial ligands seemed to affect the singlet oxygen quantum yield. Yields of 0.62 and 0.14 were achieved for **(I)** and **(II)**, respectively. The lower value obtained for **(II)** probably resulted from secondary reactions of singlet oxygen in the presence of the ruthenium complex. We also investigate how axial ligands in the ruthenium–phthalocyanine complexes affect their photo-bioactivity in B16F10 murine melanoma cells. In the case of **(I)** at 1 μmol/L, photosensitization with 5.95 J/cm^2^ provided B16F10 cell viability of 6%, showing that **(I)** was more active than **(II)** at the same concentration. Furthermore, **(II)** was detected intracellularly in B16F10 cell extracts. The behavior of the evaluated ruthenium–phthalocyanine complexes point to the potential use of **(I)** as a metal-based drug in clinical therapy. Changes in axial ligands can modulate the photosensitizer activity of the ruthenium phthalocyanine complexes.

## Introduction

In recent years, the use of metal–photosensitizer compounds in photodynamic therapy (PDT) has been proposed: such compounds allow a combinatory approach involving chemo and light irradiation therapy to be applied against cancer (Zhang et al., 2018). In this context, phthalocyanine-based complexes, which present strong absorption in the therapeutic window, good singlet oxygen quantum yield, and excellent thermal stability (Robertson et al., 2009; Liu et al., 2018), have aroused great interest and have been considered second-generation photosensitizers for use in PDT. In general, hexacoordinated phthalocyanine complexes bearing different d-metal ions can be synthesized (Liu et al., 2007; Kabwe et al., 2019; Mohammed et al., 2019; Yin et al., 2019), to afford flexible environments that could form octahedral species. To explore the structure–activity relationship of this group further, it is important to investigate the anti-cancer efficacy and specificity of new phthalocyanine complexes, which may shed light on the cytotoxicity of these compounds during clinical therapy of different types of cancer. To this end, new synthetic procedures involving template processes have emerged as a possibility to overcome the low yield and purity of metal–phthalocyanine species (Kantekin et al., 2006; Alzeer et al., 2009; Bartlett et al., 2018) that are usually obtained in reported synthetic procedures. Metal–phthalocyanine complexes (MPcs) have had their photochemical and photophysical extensively explored, but photobiological assays involving these complexes have been described less frequently (Nyokong, 2007; Lourenço et al., 2014; Ahmetali et al., 2019; Uslan et al., 2019). Insertion of heavy metals in the phthalocyanine cavity usually provides the complex with low fluorescence intensity because the intersystem crossing effect is enhanced (Tekdaş et al., 2012; Mehraban and Freeman, 2015; Matlou et al., 2019). This effect has been considered a limitation that prevents this kind of system from being applied in a theranostic approach. Understanding the fundamental aspects involved in the photo-reactivity of ruthenium–phthalocyanine complexes may support the development of new strategies to use metal-based drugs in light irradiation therapy.

Here, we present a new and potentially useful method to synthesize MPcs. Bearing in mind that ruthenium complexes containing DMSO and 4-amynopyridine ligands are toxic to cancer (Krstić et al., 2010; Brabec and Kasparkova, 2018; Angerani and Winssinger, 2019; Shum et al., 2019), we propose the synthesis of ruthenium–phthalocyanine complexes such as *trans*-chloro(dimethylsulfoxide)phthalocyanineruthenium(III) (*trans-*[RuCl(Pc)(DMSO)], or **(I)**, and *trans*-bis(4-aminopyridine)phthalocyanineruthenium(II) (*trans-*[Ru(Pc)(4-ampy)_2_], or **(II)**, and describe the effect of the molecular structure on their cytotoxicity to melanoma murine cells. In the case of B16F10 cells, **(I)** provided interesting results: at 1 μmol/L, it promoted light-induced cell death of 94.0%, whereas **(II)** reduced cell viability even in the dark.

## Experimental

### Chemicals

1,8-diazabicyclo[5.4.0]undec-7-ene (DBU), 1,2-dicyanobenzene,1,3-diphenylisobenzofuran (DPBF), 4-aminopyridine, and MTT–M5655 were purchased from Sigma-Aldrich and used as received. Calf thymus DNA was acquired from Sigma and purified by dialysis against 5 mM Tris and 50 mM NaCl (pH = 7.5) buffer three times over a 48-h period. The non-essential amino acids MEM, trypan blue solution, and culture medium RPMI 1640 were purchased from Sigma-Aldrich. Dimethylsulfoxide (DMSO), methanol, chloroform, and 1-pentanol were obtained from Synth. Ruthenium(III) chloride hydrate (RuCl_3_.nH_2_O) was acquired from Acros. Silica gel 200–400 mesh (Sigma-Aldrich) was employed for chromatographic purification. All the other reagents were purchased from Sigma and used as received.

### Equipment

The ground-state electronic absorption spectra were recorded on Agilent 8453 and Hitachi U-3501 spectrophotometers. The Raman spectra were acquired on a HORIBA JobinYvonLab Ram HR Micro Raman spectrometer (laser at 632.8 nm and 17 mW, exposure time of 80, two accumulations). The spectrum of *trans*-[Ru(Pc)(4-ampy)_2_] NMR ^1^H (400 MHz) was recorded on a BRUKER®-Model DPX400 spectrometer; DMSO-d6 was used as solvent. The mass spectra were acquired on a MALDI-TOF/TOF Ultraflextreme (Bruker Daltonics) mass spectrometer. Electronic Paramagnetic Resonance spectroscopy (EPR) was carried out on a JEOLJEOL JES-FA 200 (9.5 Hz) apparatus, at room temperature. The fluorescence emission spectra were registered on an RF-5301PC Shimadzu spectrofluorimeter. The infrared spectra were acquired on a Prestige-21 Shimadzu FTIR spectrometer (KBr pellets). The fluorescence lifetimes were measured on a MicroTime 200–PicoQuant device (time-resolved confocal fluorescence microscope with unique single molecule sensitivity). The complexes were excited with a diode laser bundle λ_ex_ = 640 nm; FWHM: 0.080-ns excitation pulse. Fluorescence was emitted, collected, and analyzed with the software SymPhoTime 5.2.4. The nanosecond transient absorption (nsTA) experiments were performed with an optical parametric oscillator (basiScan, Spectra-Physics) excitation source with pulsed Nd:YAG laser (Quanta-Ray INDI, Spectra-Physics–FWHM ~ 8 ns). The decay curves were plotted and analyzed with the software Igor Pro (6.3). The complexes were solubilized in DMSO, and the electronic spectra of all the samples were registered before and after the measurements. The irradiations were performed with Colibri Quantum Tech laser at 640 nm. Intracellular ruthenium concentrations were performed by using inductively coupled plasma optical emission spectrometry (ICP-OES, Vista-MPX CCD Simultaneous, Varian, Mulgrave, Australia).

### Synthesis of Ruthenium Complexes

#### *trans*-[RuCl(Pc)DMSO] (I)

The *trans-*[RuCl(Pc)DMSO] complex was synthesized according to methodology adapted by Adeloye and Ajibade (2014). *Cis*-[RuCl_2_(DMSO)_4_] (0.200 g, 0.41 mmol), 1,2-dicyanobenzene (0.480 g, 3.7 mmol), and 2.0 ml of DBU were added to 15 ml of previously distilled pentanol. The reaction was heated to 130°C under reflux and argon for 24 h. Subsequent addition of 100 ml of methanol afforded a blue solid, which was separated by filtration. The filtered solution was concentrated to dryness in a rotary evaporator. Next, 30 ml of toluene was added to the crude product, and the resulting solution was submitted to liquid–liquid extraction with three 20-ml portions of water. The toluene solution was concentrated to dryness in a rotary evaporator, solubilized in dichloromethane, and submitted to silica gel column chromatography. The desired complex was eluted with dichloromethane/methanol 10:1. Yield: (0.110 g, 45.0%). UV-vis (DMSO), λ_max_ (nm) (log ε): 640 (4.24), 314 (4.32). IR [(KBr) ν_max_ (cm^−1^)]: 1488 (-N=); 1411(C-H) isoindol; 1286 (C-H) in plane; 1172 (C-H) isoindol; 753 ring Pc.

#### trans-[Ru(Pc)(4-ampy)_2_] (II)

*Trans-*[Ru(Pc)(4-ampy)_2_] was synthesized by adapting the method described by Rawling et al. (2007). First, **(I)** (0.100 g, 0.16 mmol) and 4-aminopyridine (0.046 g, 0.48 mmol) were added to chloroform. The resulting mixture was kept under reflux at 60°C for 6 h, and the resulting solution was cooled to room temperature. Methanol/water 3:1 was added, and the precipitate was collected under vacuum filtration. The solid was successively washed with water and methanol and dried under vacuum. Yield: (0.01 g, 10%). UV-vis (DMSO), λ_max_ (nm) (log ε): 623 (4.72), 385, 317 (4.82). IR [(KBr) ν_max_ (cm^−1^)]: 3452 (N-H); 1511 (C=C) aromatic; 1488 (-N=); 1414(C-H) isoindol; 1291 (C-H) in plane; 1172 (C-H) isoindol; 753 ring Pc. MALDI-TOF MS m/z calc. 802.160, found 802.172.

### Photophysical Studies

#### Fluorescence Quantum Yields

The fluorescence quantum yields of **(I)** and **(II)** were calculated by a comparative method (D'Souza et al., 2011). Phthalocyanine zinc(II) [Zn(Pc)] (Φ_fstd_ = 0.2 in DMSO) was used as standard. Excitation was conducted at 610 nm, slit 3, and Equation 1 was employed.

(1) $$\begin{array}{r} \Phi_{f}=\Phi_{fstd}\cdot \frac{F\cdot A_{std}\cdot n^{2}}{F_{std}\cdot A\cdot n_{std}^{2}} \end{array}$$

where Φ_fstd_ is the fluorescence quantum yield of the standard; F_std_ and F are the area under the curve of the fluorescence spectrum of the standard and the sample, respectively; A_std_ and A are the area of the absorbance band of the standard and the sample at the excitation wavelength, respectively; and n^2^ is the refractive index of the solvent.

### Photochemical Studies

#### Singlet Oxygen Quantum Yields

The singlet oxygen quantum yields of **(I)** and **(II)** were quantified by an indirect method that used 1,3-diphenylisobenzofuran (DPBF) as singlet oxygen acceptor (Tekdaş et al., 2012). Briefly, 2 ml of a solution of **(I)** or **(II)** was prepared in DMSO, so that absorbance of about 0.3 at 660 nm was achieved. [Zn(Pc)] was used as reference, and DPBF was employed as chemical quencher for singlet oxygen. The singlet oxygen quantum yield was calculated on the basis of Equation 2:

(2) $$\begin{array}{r} \Phi_{\Delta }=\Phi_{\Delta std}\cdot \frac{R\cdot I_{std}^{abs}}{R_{std}\cdot I^{abs}} \end{array}$$

where Φ_Δ*std*_ is the singlet oxygen quantum yield for the standard ZnPc (Φ_Δ*std*_ = 0.67 in DMSO); R and R_std_ are the photobleaching rates after irradiation of **(I)** or **(II)** and the standard, respectively; and I^abs^ and ${\text{I}}_{\text{std}}^{\text{abs}}$ are the rates of light absorption by **(I)** or **(II)** and the standard, respectively.

#### Photodegradation Studies

The photodegradation studies were accomplished by preparing solutions of **(I)** or **(II)** in DMSO, in order to obtain Q band absorbance between 1.0 and 1.5. First, the spectra were registered; then, the samples were irradiated with light at 640 nm and power of 39 mW for 5 min. The spectra were registered after each irradiation. For each sample, between five and eight irradiations were performed.

### Cytotoxicity Studies

#### Cell Culture

The *in vitro* cytotoxicity of *trans*-[Ru(Pc)(4-ampy)_2_] (complex **II**) against B16F10 murine melanoma cells in culture was investigated. B16F10 cells were cultured in RPMI-1640 medium (R6504, Sigma-Aldrich) supplemented with 10% fetal bovine serum and antibiotic antimycotic solution (A5955, Sigma-Aldrich) containing 100 U/ml penicillin G, 0.1 mg/ml streptomycin, 0.25 μg/ml amphotericin B, and 2 g of sodium bicarbonate, pH 7.4. The cells were cultured in 75-cm^2^ tissue culture flasks in a humidified incubator at 37°C with 5% CO_2_ until 75–90% confluence was achieved.

#### MTT Cell Viability Assay

B16F10 cell viability was assessed after treatments with **(I)**, **(II)**, or vehicle (control condition; 1% DMSO); the thiazolyl blue tetrazolium bromide (MTT, M5655, Sigma-Aldrich) colorimetric assay was used. The cells were plated on 96-well cell culture plates at 1 × 10^5^ cells/well for 24 h. The viability assays were conducted at different concentrations, with and without light irradiation (λ = 660 nm; dose = 5.95 J/cm^2^). After the treatments, the cells were incubated with MTT solution (0.5 mg/ml) at 37°C for 3 h The MTT solution was discarded, and the cells were exposed to 100% DMSO at room temperature for 1 h. The absorbance was measured at 492 nm with a multi-well plate reader. Cell viability was expressed as the percentage of the absorption values in the cells treated with **(I)** or **(II)** relative to the vehicle (control) cells.

#### Flow Cytometry Analysis

The cell death mechanism was evaluated by flow cytometer; the Annexin V-FITC Apoptosis Detection Kit (APOAF; Sigma-Aldrich) was used. B16F10 cells were incubated with **(II)** at 1 μmol/L for 24 h, with and without light irradiation (λ = 660 nm; dose = 5.95 J/cm^2^). Then, the cells were incubated at 37°C and 5% CO_2_ for 24 h. After treatment, the cells were submitted to trypsinization and stained with 5 μl of annexin V-FITC and 1 μl of each sample (sample concentration = 100 μg/ml). The cells were then incubated at room temperature for 15 min, 400 μl of 1 × annexin-binding buffer was added, the mixture was mixed gently, and the samples were kept on ice. For flow cytometry analysis, the cells were exposed to 2% propidium iodide solution. All the cells were analyzed by flow cytometry (FACS Canto™ II, BD Biosciences; FAPESP #04/09448-5). The fluorescence emission at 530/30 nm and 695/40 nm, with Argon laser 488 nm, was measured. The data were analyzed by using the software BD FACSDiva™.

#### Statistical Analysis

The data are presented as the mean ± standard error mean (S.E.M.) of at least three independent experiments (*n*), performed in triplicate for each experimental *n*. Statistical analysis was performed by two-way ANOVA, followed by Bonferroni *post hoc*. Results with *P* < 0.05 were considered statistically significant.

## Results

### Characterization of Ruthenium–Phthalocyanine Complexes

**Figure 1:**
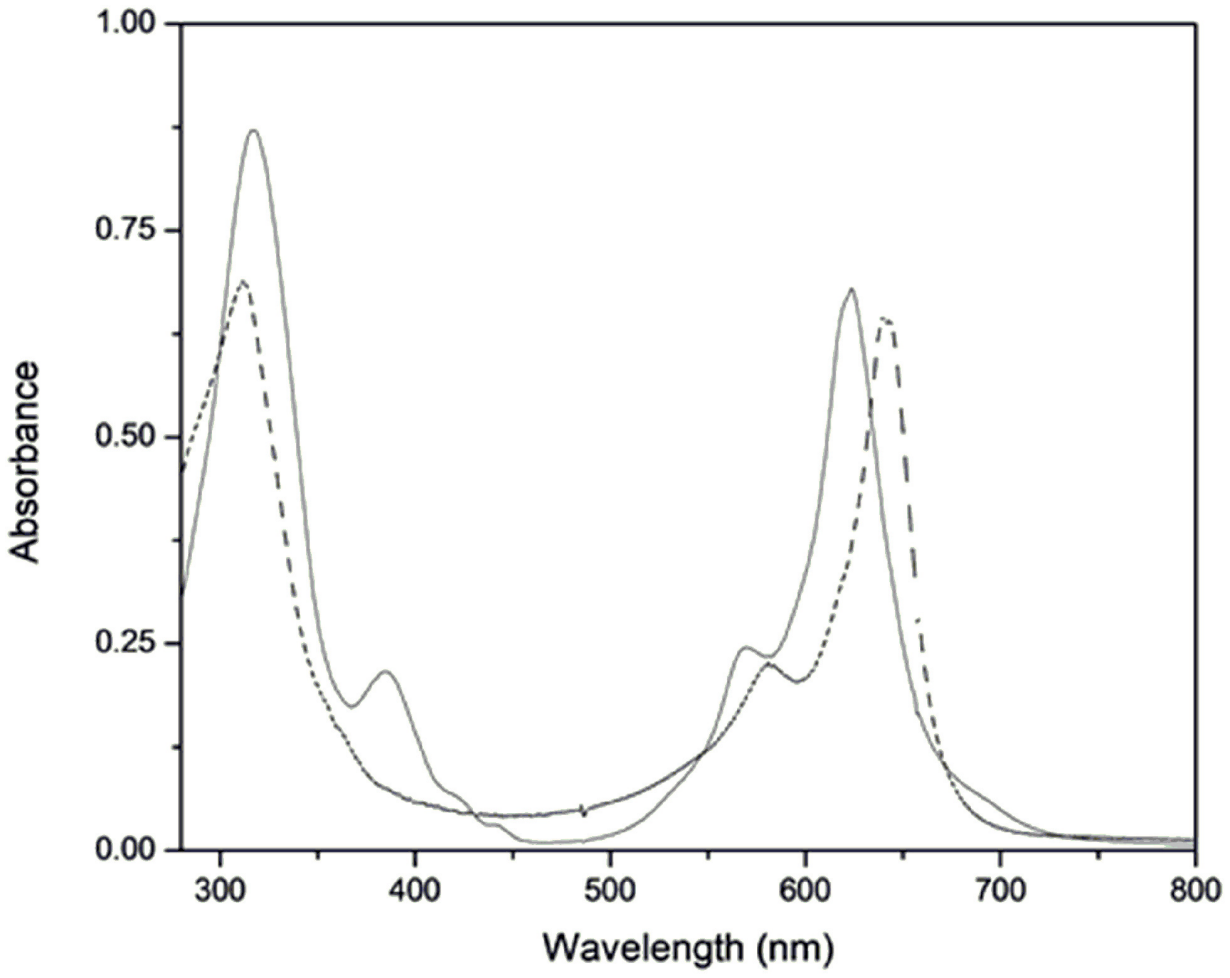
Ground-state electronic spectra of **(I)** (solid line) and **(II)** (dashed line) at 1.3 × 10^−5^ mol L^−1^ in DMSO.

We characterized the new ruthenium–phthalocyanine compounds **(I)** and **(II)** by MALDI-TOF mass spectrum, UV-vis, FTIR, EPR, Raman Resonance, and NMR. The MS MALDI-TOF spectra showed a pseudo-molecular ion peak for **(I)** and **(II)**, which agreed with the proposed molecular formula. For **(I)**, we spotted peaks with *m/z* = 614.248 and 1226.092; for **(II)**, the spectrum showed main peaks with *m/z* = 614.056, 802.172, and 1227.102 (Supplementary Figures 1, 2). We assigned all the major peaks and considered the most abundant ruthenium isotope. The ground-state UV-vis spectra of **(I)** and **(II)** were typical of phthalocyanine-like species. Figure 1 shows the UV-vis absorption spectra of **(I)** and **(II)**.

The electronic spectrum of complex **(I)** presented a Q-band at 640 nm (log ε = 4.70) and a Soret band at 316 nm (log ε = 4.72). The electronic spectrum of **(II)** exhibited a blue-shifted Q-band at 623 nm (log ε = 4.72) and a Soret band at 317 nm (log ε = 4.82). The electronic spectrum of **(II)** also had an absorption band at 385 nm. We observed the Q-band vibronic coupling (Palys et al., 1994; Claessens et al., 2008; Ogunsipe and Nyokong, 2009) in the spectra of **(I)** and **(II)** as a shoulder at 581 and 568 nm, respectively. The FTIR spectra of **(I)** and **(II)** presented vibrational modes at 1,488, 1,411, and 753 cm^−1^, which were characteristic of phthalocyanine ring (data not shown). For **(II)**, the absorption band at 3,452 cm^−1^ indicated the presence of 4-aminopyridine as axial ligand.

**Figure 2:**
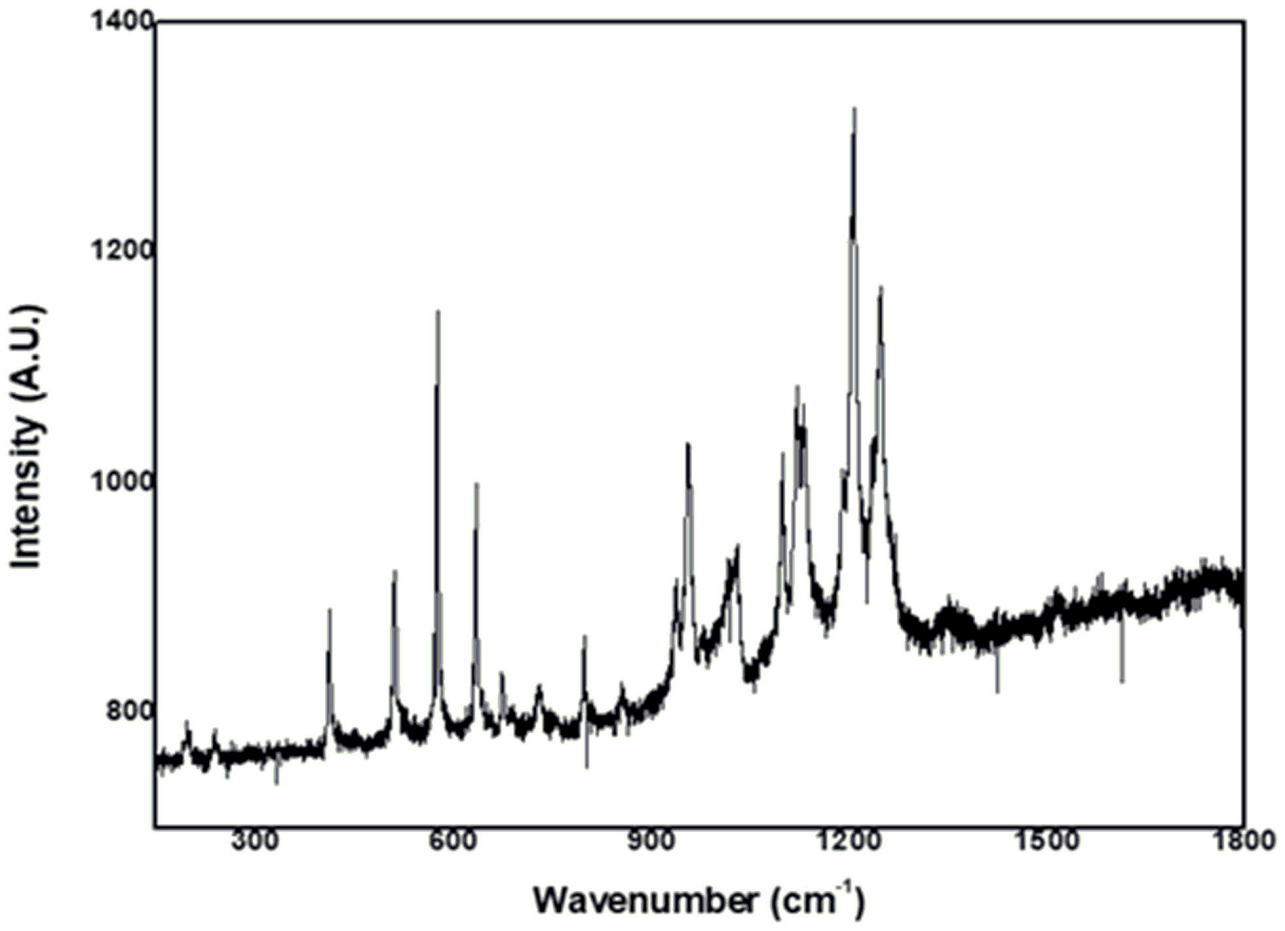
Resonance Raman spectrum of *trans-*[Ru(Pc)(4-ampy)_2_].

We obtained enhanced Raman Resonance with weak signals for both complexes (Figure 2 and Supplementary Table 1). For **(II)**, we observed some bands attributed to the phthalocyanine ring (1,414, 1,120, 954, and 730 cm^−1^) and a band at 239 cm^−1^, described as being due to the metal–nitrogen vibration mode.

**Figure 3:**
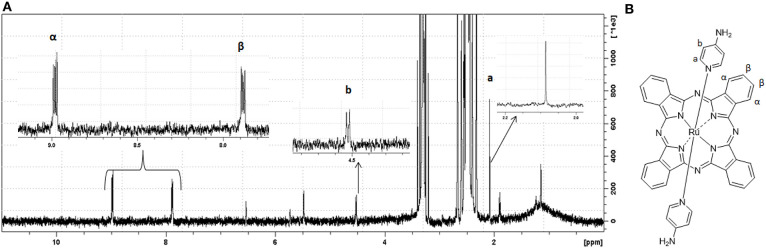
^1^H NMR and suggested molecular structure of *trans*-[Ru(Pc)(4-ampy)_2_]. In **(A)**, ^1^H NMR spectrum of complex **(II)** in DMSO-d6. In **(B)**, suggested molecular structure of complex **(II)** poiting to specific ppm range hydrogens.

The ^1^H NMR spectra of **(I)** and **(II)** displayed proton signals that were consistent with multiplets of the phthalocyanine aromatic ring, –β H between 8.99 and 8.96 ppm and –α H between 7.90 and 7.86 ppm (Figure 3 and Supplementary Figure 3). For **(I)**, we observed a proton signal at −1.18 ppm. The ^1^H NMR spectrum of **(II)** presented proton signals due to the axial ligands 4-aminopyridine, –H_b_ δ = 4.53 ppm and H_a_ δ = 2.08 ppm.

### Photophysical and Photochemical Properties

We analyzed the photochemical and photophysical parameters of **(I)** and **(II)** to understand how these photosensitizer candidates behave. Table 1 summarizes the singlet oxygen quantum yields (Φ_Δ_), fluorescence quantum yields (**Φ_f_**), and singlet fluorescence lifetime (τ_**1**_ and τ_**2**_) of both complexes. These yields were obtained at low concentration of the complexes to avoid aggregation.

**Table 1:** Photochemical and photophysical parameters of complexes **(I)** and **(II)**.

	**Φ_Δ_**	**Φ_f_**	**τ_1_ (ns)**	**α_1_/%**	**τ_2_ (ns)**	**α_2_/%**
Complex (I)	0.62	0.007	1.8	68.2	3.2	31.8
Complex (II)	0.14	0.011	1.9	23.3	3.1	76.7

**Figure 4:**
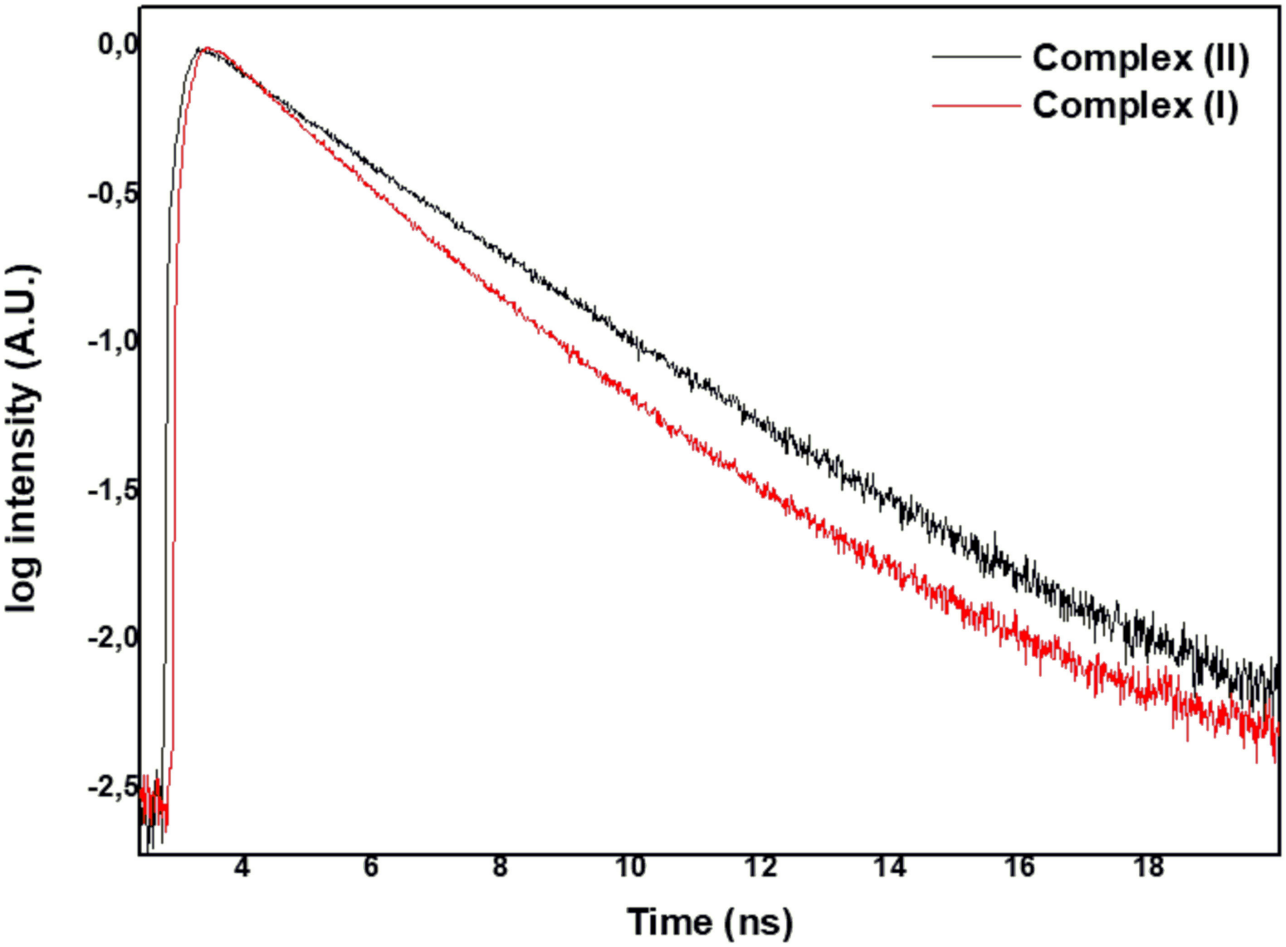
Fluorescence curve decays for (**I**) and (**II**).

The measured fluorescence intensity decay profiles of **(I)** and **(II)** related to the emission of the S_1_ → S_0_ transition showed similar trends. Figure 4 presents the temporal profiles recorded at 670 nm following photoexcitation of **(I)** and **(II)** at 640 nm in DMSO solution.

The curve in Figure 4 fit a bi-exponential curve well, and the output provided two fluorescent lifetimes: the short one around 1.85 ns and the longer one around 3.20 ns (Table 1).

**Figure 5:**
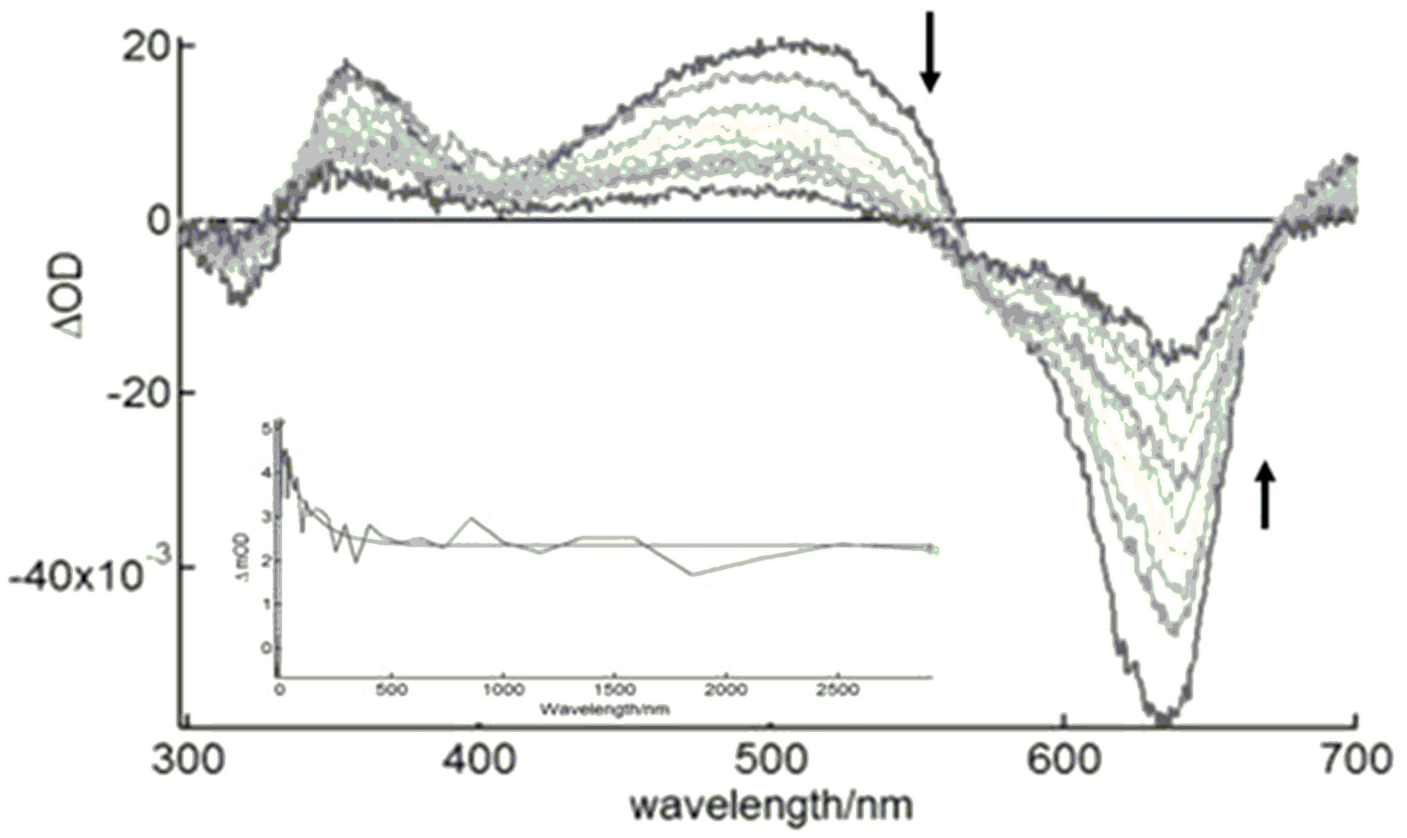
Nanosecond TA spectra of **(I)** in deaerated DMSO after pulsed laser excitation (λ_ex_ = 620 nm).

We measured the excited triplet-state transient time profiles of **(I)** in deaerated DMSO in fluid solution at 25°C by using nanosecond laser flash photolysis at 620 nm. The features of the transient absorption spectra revealed that the low-energy absorption band, described as Q-band, diminished, whereas new absorption bands appeared. Typical transitions originating from T_1_ to T_2_ and upper triplet states (T_1_ to T_n_) were evident for **(I)**—Figure 5—as observed in a similar system (Anula et al., 2006). The triplet–triplet absorption bands emerged at 520 and 360 nm. We also noted that the triplet excited state formed at the 700-nm region, but this was not completely clear due to the wavelength range. The triplet state lifetime fit a bi-exponential curve well (Figure 5). It comprised a fast process with a time constant of 15 ns, which suggested a triplet–triplet recombination process detected in highly concentrated metal–phthalocyanine solutions (Debacker et al., 1988; Nwaji and Nyokong, 2017) or probably a process of aggregation of excited states (FitzGerald et al., 2002). A slower process with a time constant of about 3.68 ± 0.02 μs was consistent with the triplet state of ruthenium phthalocyanines (Ferraudi and Prasad, 1984).

### Photobiological Properties

**Figure 6:**
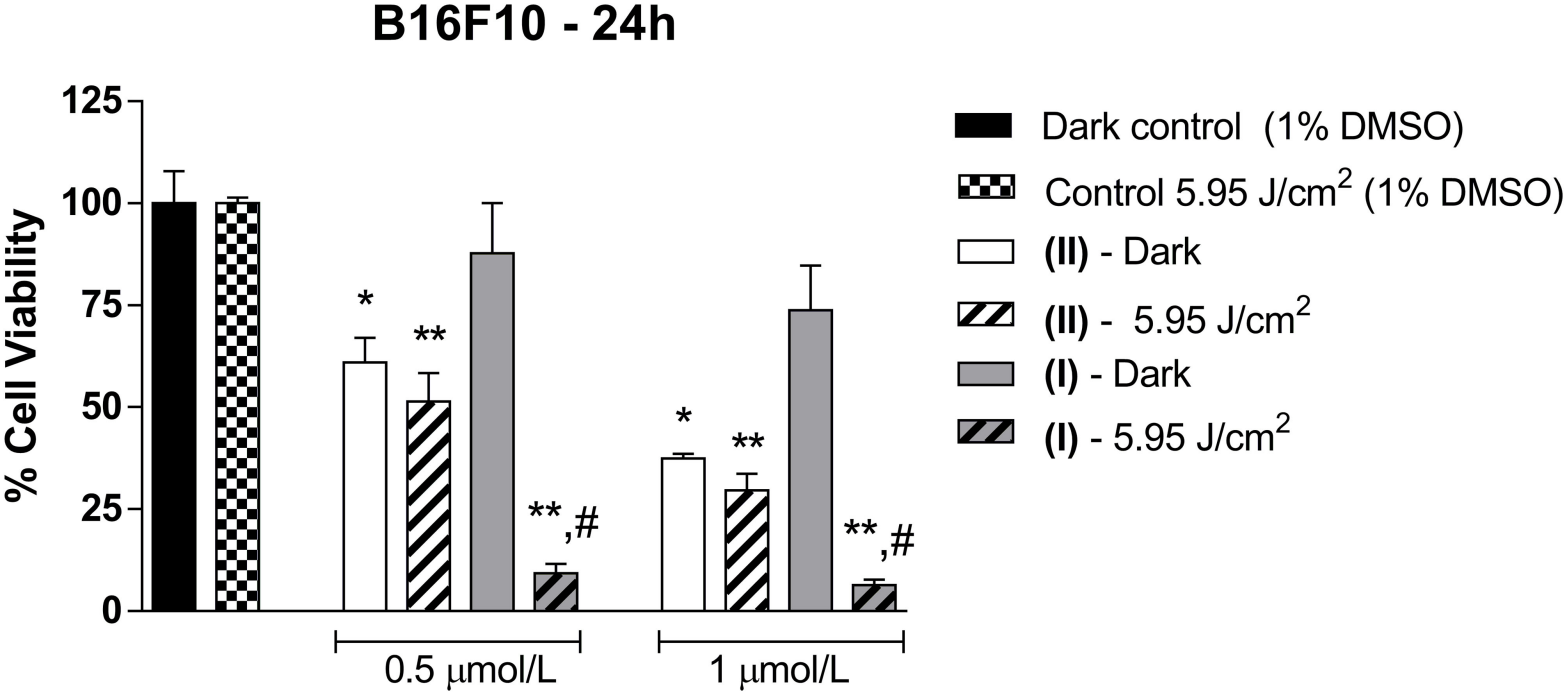
B16F10 cell viability in the presence of **(I)** or **(II)**. The cells were incubated with different concentrations of **(I)** or **(II)**. (0.5 or 1 μmol/L) for 24 h and were irradiated with 660-nm light at 5.95 J/cm^2^. Data are the mean ± S.E.M. Two-way ANOVA, with Bonferroni *post-hoc* (*P* < 0.05). *different from Dark control; **different from Control under 5.95 J/cm^2^; ^**#**^different from **(I)** dark. All the results in this figure are representative of independent experiments (*n* = 3) performed in triplicate for each experimental *n*.

We investigated the viability of B16F10 murine melanoma cells in the dark or following irradiation with 660-nm light at 5.95 J/cm^2^ in the presence of **(I)** or **(II)** at 0.5 or 1 μmol/L. Figure 6 shows the B16F10 cell viability.

The presence of **(I)** only decreased B16F10 cell viability after light irradiation at 660 nm, independent of the concentration of **(I)** [0.5 μmol/L of **(I)**: 9.32 ± 4.00%; and 1 μmol/L of **(I)**: 6.43 ± 2.38%; *n* = 3] compared to dark (100.01 ± 13.66%; *n* = 3) and control light (100.03 ± 2.40%; *n* = 3) conditions (Figure 6). However, **(I)** did not exhibit significant effects in the dark [0.5 μmol/L of **(I)**: 87.80 ± 21.24%; and 1 μmol/L of **(I)**: 73.81 ± 19.00%; *n* = 3].

Figure 6 also shows B16F10 cell viability after treatment with **(II)** for 24 h. Compared to the control condition, B16F10 viability in the presence of **(II)** reduced in the dark [0.5 μmol/L of **(II)**: 60.97 ± 12.20%; and 1 μmol/L of **(II)**: 37.41 ± 2.11%; *n* = 3–4] or after light irradiation [0.5 μmol/L of **(II)**: 51.37 ± 14.00%; and 1 μmol/L of **(II)**: 29.62 ± 8.09%; *n* = 4] at both concentrations of **(II)**. Increasing the concentration of **(II)** to 2 or 4 μmol/L boosted its cytotoxicity effect (Supplementary Figure 4). In addition, after B16F10 cells were treated with **(II)**, ruthenium was detected intracellularly in cellular extracts (0.8584 ng/μl), as observed in Supplementary Table 2, but this metal was not detected in control cellular extracts.

**Figure 7:**
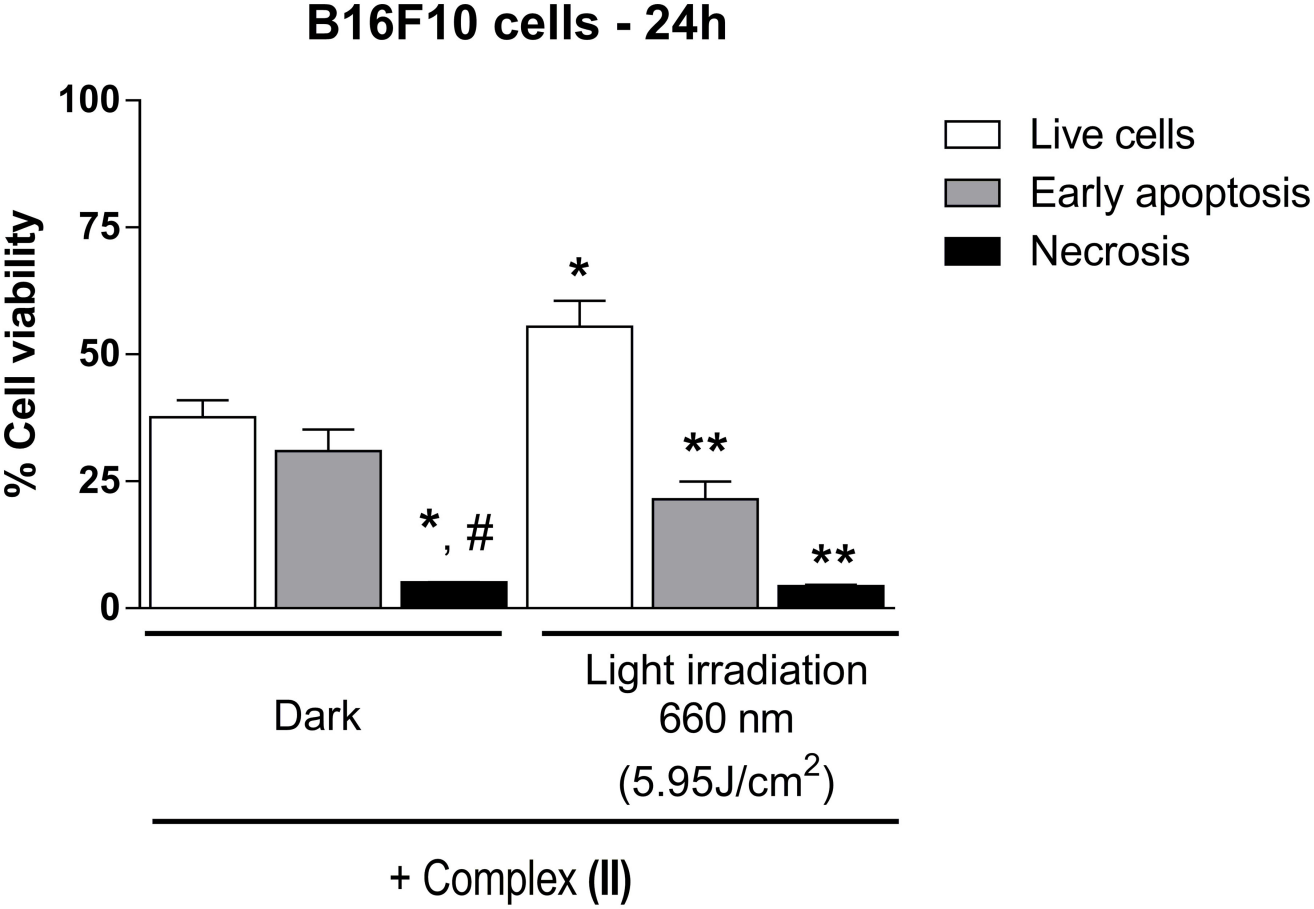
Apoptosis and necrosis evaluation in B16F10 cells in the presence of **(II)**. B16F10 cells, in culture, were exposed to **(II) (**1 μmol/L, 24 h) in the culture medium, in the dark or under 660-nm light irradiation (5.95 J/cm^2^). Data are presented as mean ± S.E.M. Two-way ANOVA followed by Bonferroni *post-hoc* (*P* < 0.05); *n* = 3. *different from respective Live cells Dark; **different from Live cells Light irradiation 660 nm; ^**#**^different from Early apoptosis Dark.

We also investigated the cell death mechanism of B16F10 cultured cells in the dark condition or under 660-nm light irradiation after the cells were treated with **(II)** for 24 h (Figure 7).

In the presence of **(II)** and in the dark condition, there were early apoptosis (30.97 ± 4.21%; *n* = 3) and necrosis (5.1 ± 0.06%; *n* = 3) activation compared to live cells (37.60 ± 3.33%; *n* = 3). In addition, after light irradiation at 660 nm (5.95 J/cm^2^), B16F10 cells also presented early apoptosis (21.47 ± 3.52; *n* = 3) and necrosis (4.4 ± 0.25; *n* = 3) compared to live cells (55.43 ± 5.09; *n* = 3). Both **(I)** and **(II)** could exert cytotoxicity effects by interacting with DNA, which could somehow contribute to the biological mechanism related to cancer cell death. Therefore, we evaluated the interaction of **(I)** or **(II)** with DNA by UV-visible spectroscopy. A typical UV-visible spectrum of **(I)** with Calf Thymus (CT) DNA is shown in Supplementary Figure 5. While the Q-band of **(I)** decreased with increasing CT DNA concentration, the spectroscopic bands of **(II)** did not change after CT DNA supplementation, suggesting that DNA did not interact with **(II)**.

## Discussion

Metal–phthalocyanine synthesis is key to improving the use of these complexes in PDT (Allison et al., 2004; Boyar and Çamur, 2019; Fujishiro et al., 2019). Among such complexes, ruthenium–phthalocyanine has emerged as a promising molecule because ruthenium complexes generally have octahedral geometry, and ruthenium–phthalocyanine is a hexacoordinated species that can still bind two new axial ligands (Heinrich et al., 2014; Teles Ferreira et al., 2017). The main concern involving ruthenium–phthalocyanine complexes is their synthesis and difficulties related to impurities are frequently reported. Previous communications described that *cis*-[RuCl_2_(DMSO)_4_] is the precursor for the preparation of many substituted and unsubstituted phthalocyanines (pc-R) (Adeloye and Ajibade, 2014). The synthesis of [Ru(pc-R)] comprises steps such as addition of phthalonitrile and appropriate reagents to *cis*-dichlorotetrakis(dimethylsulfoxide)ruthenium(II) in dimethylformamide and heating under reflux for several hours. We followed a similar procedure to prepare ruthenium–phthalocyanine, but we used 1-pentanol as solvent. The spectroscopic results suggested that our final product composition differed from the final product composition reported in the literature. The electrophoretic mobility gel measured for the ruthenium–phthalocyanine complex synthesized in this work did not show any movement toward applied positive or negative charge, which was consistent with a neutral species (data not shown).

The ^1^H-NMR spectrum of the ruthenium–phthalocyanine complex prepared here displayed broad multiplets in the 7–9 ppm region, which corresponded to the macrocycle aromatic portion (Kobel and Hanack, 1986; Rawling and McDonagh, 2007). The most intriguing ^1^H NMR signal emerged at −1.15 ppm (Supplementary Figure 3) and had been observed for the ruthenium–phthalocyanine complex previously synthesized by following the Kobel and Hanack method (Kobel and Hanack, 1986). Comparison of our NMR data with the ^1^H NMR of *trans*-[Ru(pc)(DMSO)_2_] (Kobel and Hanack, 1986) allowed us to detect a similar signal at −1.18 ppm, which had previously been attributed to the methyl group of coordinated dimethylsulfoxide. Therefore, we suggested that dimethylsulfoxide coordinated to the ruthenium ion in the ruthenium–phthalocyanine complex prepared herein. Although some ruthenium(III) complexes can exhibit changes in NMR signals due the paramagnetic metal, ruthenium(III) phthalocyanines can show no significant changes in NMR signals (Guo et al., 2012).

**Scheme 1:**
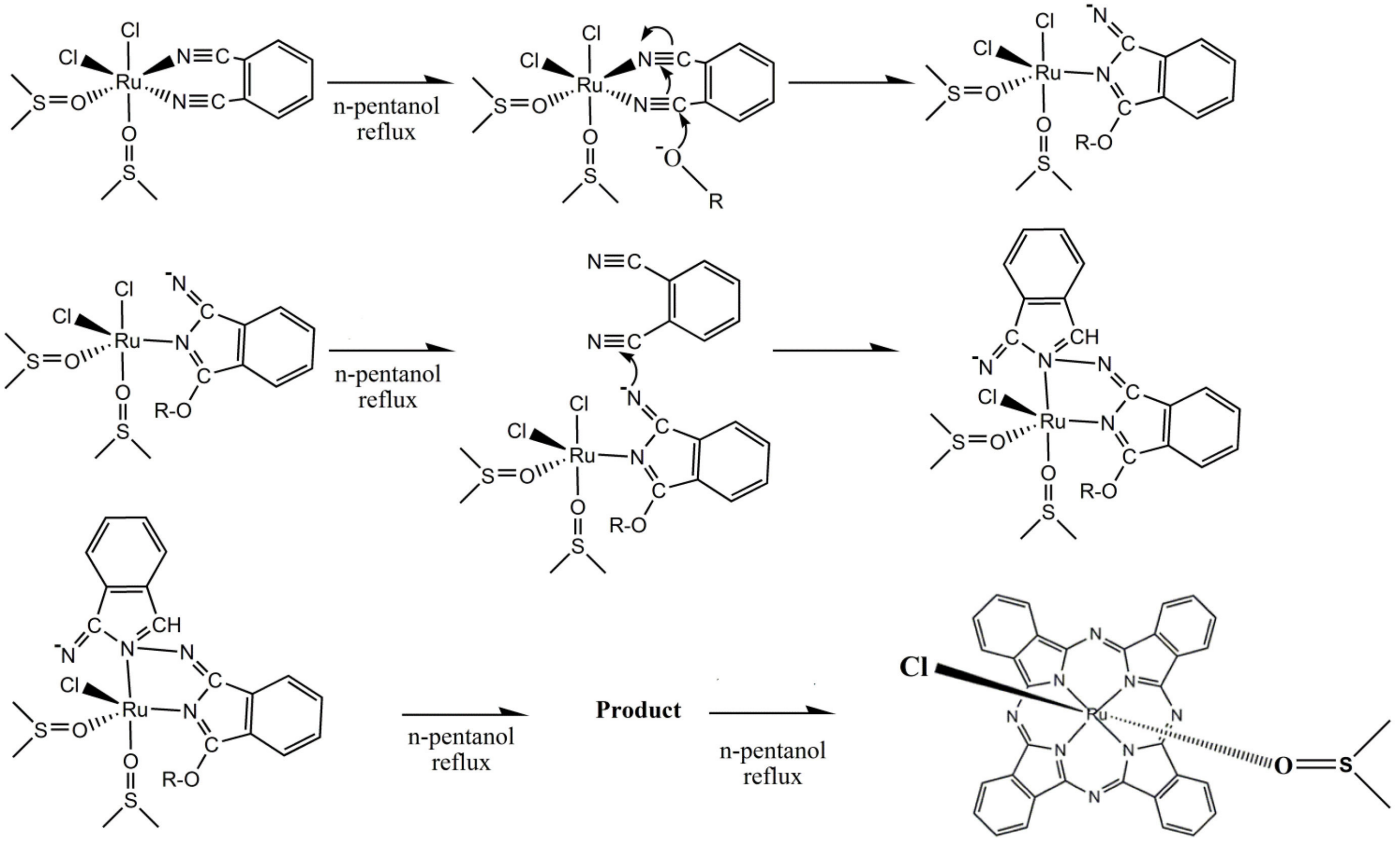
Mechanism of the synthesis of **(I)**.

We also used MALDI-TOF mass spectrometry to investigate the structure of the ruthenium–phthalocyanine complex (Supplementary Figure 1). We observed relevant peaks ascribed to [Ru(pc)]^+^ (*m/z*= 614.248) and {[Ru(pc)]_2_}^+^ (*m/z*= 1226.092), respectively (most abundant ruthenium isotope). A previous report had also described these species for axial ruthenium–phthalocyanine complexes (Rodríguez-Morgade et al., 2009). The EPR spectra of the ruthenium–phthalocyanine complex synthesized here indicated the same pattern that is usually verified for Ru^3+^ species (Khan et al., 1990) (Supplementary Figure 6). The complex exhibited three lines with different “g” values, g_x_ = 2.53, g_y_ = 2.57, and g_z_ = 2.54. The neutral character of the prepared ruthenium–phthalocyanine complex evidenced by gel electrophoresis suggested coordination of a monoanionic ligand, which we inferred as being chloride because we used *cis*-[RuCl_2_(DMSO)_4_] as precursor. Taken together, these results indicated that the ruthenium–phthalocyanine complex synthesized in this work could be better described as **(I)**. Despite the suggestion, we were not able to identify **(I)** by MALDI-TOF unambiguously probably because axial ligands were lost. The overall reaction for the synthesis of **(I)** could be summarized as a template process during which the ruthenium ion governs macrocycle cyclization (Scheme 1). Isolation of [RuCl_2_(DMSO)_2_(phthalonitrile-R)] as intermediate for the synthesis of asymmetric ruthenium–phthalocyanines (Negri et al., 2018) reinforced the reaction mechanism depicted in Scheme 1.

**(I)** was a versatile starting material for [Ru(pc)L_2_] species. Therefore, we prepared **(II)** to understand how 4-aminopyridine (4-ampy) as axial ligand affected cytotoxicity. In time, 4-ampy has been used as an antiproliferative agent in cancer cells (Woodfork et al., 1995; Wang et al., 2011; Hassan et al., 2018), and it may add some biological properties to the ruthenium–phthalocyanine complex. *trans*-[Ru(Pc)(4-ampy)_2_] was EPR silent, which suggested that free 4-ampy reduced **(I)** during the synthesis (Díaz-García et al., 2009). The MS MALDI-TOF spectrum showed main peaks (most abundant ruthenium isotope) that agreed well with the molecular formula of [Ru(pc)]^+^ (*m/z* = 614.056), {[Ru(Pc)(4-ampy)_2_]^+^} (*m/z*= 802.172), and {[Ru(pc)]_2_]^+^}(*m/z*= 1227.102) (Supplementary Figure 2) (Rodríguez-Morgade et al., 2009). The ^1^H NMR spectrum presented proton signals that were assigned to the axial ligands, which were shifted to higher field (H_b_ δ = 4.53 ppm and H_a_ δ = 2.08 ppm—see Figure 3) as compared to free 4-ampy, as also reported by Rawling et al. (2007) for the ^1^H NMR spectra of tetrasubstituted ruthenium–phthalocyanine complexes coordinated with 4-ampy ligands. The authors commented that the shifts were probably due to the proximity of the axial ligands with the diamagnetic ring and that the major shift in H_a_ signals was consistent with the proximity of these protons with the Pc ring. The well-resolved ^1^H NMR spectrum revealed a diamagnetic molecule profile.

The general UV-visible spectrum of phthalocyanine complexes presents particular bands in the visible region, attributed to electronic transitions from HOMO (the highest occupied molecular orbital) to LUMO (the lowest unoccupied molecular orbital). The ground-state electronic spectra of free phthalocyanine (H_2_Pc) and MPcs generally differ significantly: H_2_Pc displays two bands between 600 and 700 nm and two other bands between 290 and 350 nm as a result of their lower symmetry. Metal insertion promotes higher symmetry and causes degeneracy in LUMO, so only one band can be observed in each of the aforementioned regions (Lever et al., 1981; Claessens et al., 2008; Van Leeuwen et al., 2014; Martynov et al., 2019). We verified these characteristics in the electronic spectra of **(I)** and **(II)** (Figure 1). Compared to the electronic spectrum of **(I)**, the electronic spectrum of **(II)** exhibited blue-shifted Q- and Soret bands, which suggested that coordination of the axial ligands modified the molecular orbitals. The electronic spectrum of **(II)** also had an absorption band at 385 nm, which we ascribed to metal ligand charge transfer (MLCT) dπ_(Ru)_-π^*^(4-ampy) by comparison with a similar species (Rawling et al., 2007; Heinrich et al., 2014).

We recorded enhanced Raman Resonance spectra for **(I)** and **(II)** and correlated them with the respective UV-visible spectrum. Analysis of both complexes showed two different features. When excited at 632.8 nm, **(I)** presented very weak Raman Resonance (RR) signals, which made attribution impossible. We speculate that the reason for this could be the existence of chromophores of the molecule that are not involved in the resonance Raman enhancement of a particular vibrational mode, which should attenuate the Raman intensities (Hong and Asher, 2015). Excitation at 632.8 nm selectively enhanced the phthalocyanine vibration modes for **(II)** (Figure 2), which essentially showed that there was no molecular orbital contribution from 4-aminopyridine to the band located in the 660-nm region. We identified the nature of the individual RR bands of **(II)** and describe them in Supplementary Table 1. We ascribed the weak band at 239 cm^−1^ to the Ru-N(Pc) bond, and this band attested that the metal orbitals contributed to the composition of the Q-band.

We also investigated the photophysical and photochemical properties of **(I)** and **(II)** in solution. Upon excitation at 610 nm, the fluorescence bands of these complexes arose in the region of 670 nm. Table 1 lists the fluorescence quantum yields and lifetime decays of **(I)** and **(II)**. The low values of fluorescence quantum yields were consistent with heavy metal–phthalocyanines spin-orbit coupling favored intersystem crossing (Guo et al., 2012). Furthermore, we observed two lifetimes, which indicated two different molecular populations: τ_1_ was related to changes in the vertical of the metal ion in relation to the planar aromatic ring, and we detected a metal-free phthalocyanine emission (Guo et al., 2012). The second lifetime τ_2_ was typical of metal–phthalocyanine compounds (Vincett et al., 1971; Byun et al., 2019; Sen et al., 2019). Ruthenium orbitals mixed with the molecular orbital of phthalocyanine to some degree, favoring the non-radiative deactivation process.

Photobleaching studies of **(I)** and **(II)** provided fascinating results (Supplementary Figure 7). Both complexes exhibited sluggish photodegradation kinetics as judged from the unchanged UV-visible spectra in DMSO solution even after irradiation for 40 min. This suggested that the ground-state complex or triplet oxygen might not attack the excited state of the ruthenium–phthalocyanine complexes under irradiation at 660 nm, as opposed to what is generally observed for phthalocyanine derivative complexes (Idowu and Nyokong, 2009; Demirbaş et al., 2019; Güzel et al., 2019). Molecular orbital mixing between phthalocyanine and Ru(II) or (III) might have stabilized the phthalocyanine cycle due to the high activation energy process involving attack by singlet oxygen. We evaluated the efficiency of the potentially new ruthenium-based PDT agents by singlet-oxygen quantum yield (Table 1). **(I)** was an efficient photosensitizer for singlet oxygen production, while **(II)** showed an opposite behavior. **(I)** had around four times more efficient singlet oxygen production than **(II)**. This difference could be due to the fast nonradiative decay of excited states or perhaps to the fact that 4-ampy suppressed the singlet oxygen quantum yield in **(II)** (Kearns, 1971; Ushakov et al., 2015; Petit et al., 2019). Al-Nu'airat and co-workers (Al-Nu'Airat et al., 2017) have recently described the well-known singlet oxygen scavenging character of amine groups in the photo-oxidation of aniline. This process was attributed to the singlet oxygen energy transfer process. At this point of our work, the nature of the products involving auto-oxidation of **(II)** by singlet oxygen is not clear.

By analyzing the photobiological properties of **(I)** and **(II)** on the basis of the viability of B16F10 murine melanoma cells (Figure 6) and by considering complex **(I)** as a photosensitizer, our results corroborated with the findings of Maduray and Odhav (Maduray and Odhav, 2013), who used gallium (GaPcCl), indium (InPcCl), or iron (FePcCl) phthalocyanine chlorides as photosensitizers in PDT on human lung adenocarcinoma cells (A549). In the latter case (Maduray and Odhav, 2013), compared to the control, 2 μg/ml GaPcCl, InPcCl, or FePcCl decreased A549 viability to 30, 36, and 49%, respectively, after 661-nm light irradiation at 2.5 J/cm^2^.

In contrast to the results obtained after treatment of B16F10 cells with **(I)**, treatment of these cells with **(II)** induced cytotoxicity even in the dark, probably because the axial ligands (4-ampy) in **(II)** interacted with cell components. The ligand 4-ampy is known as a non-selective voltage-gated potassium channel (K_V_) blocker (Renaudo et al., 2004), and this ligand in the axial position of **(II)** might interact with these channels in the B16F10 cell plasma membrane. In addition, the channel subtype K_V_1.3 has been found in the plasma membrane of human melanoma cells (Artym and Petty, 2002), and 4-ampy can block K_V_ in T-leukemic Jurkat cells, decreasing cell growth and proliferation by accumulating cyclin-dependent kinase inhibitor p27^kip1^ with low effects on apoptotic mechanisms (Renaudo et al., 2004). Therefore, it is evident that K^+^ channel regulation could affect cell proliferation, and **(II)** might be retained in plasma membrane in association with K_V_. However, this putative interaction could minimize the effects of **(II)** on B16F10 cells, impairing its singlet oxygen production (see Table 1) and hence its cytotoxic action as a photosensitizer. We investigated the mechanism of B16F10 cell death in the presence of **(II)** in a flow cytometry experiment (Figure 7). **(II)** seemed to induce a weaker apoptosis mechanism after 660-nm light irradiation than in the dark condition, but this was not statistically significant. Furthermore, the value of live cells after 660-nm light irradiation was higher than in the dark condition. This could be related to an impaired action of **(II)** as a photosensitizer due to its interaction with K_V_ channels in B16F10 cell plasma membrane.

Our results about the interaction of the complexes with CT DNA suggested that **(I)** could interact with DNA, as judged from the Q-band hypochromism in the UV-vis spectra, which pointed out an intercalation between this compound and DNA. Similar results have been observed for planar aromatic molecules that can interact by π-π stacking interactions with DNA base pairs (Sirajuddin et al., 2012, Sirajuddin et al., 2013). However, further experiments are needed to verify this interaction. Contrary to **(I)**, **(II)** did not interact with CT DNA, so we can suggest that B16F10 cell death could be related with K_V_ channels in B16F10 cell plasma membrane, which impaired the action of **(II)** as a photosensitizer, leading to low apoptosis and weak cytotoxicity effects that do not depend on interaction of **(II)** with DNA.

In conclusion, the adapted methodology we used to synthesize **(I)** and **(II)** is advantageous in terms of purification, providing the complexes in good yields. The photochemical and photophysical properties of **(I)** and **(II)** revealed interesting characteristics including photostability, which indicated that these compounds could be photosensitizer candidates. Insertion of different axial ligands modified some properties like singlet oxygen quantum yields and cytotoxicity effects. Results of the cytotoxicity assays attested to the high cytotoxicity of **(I)** under irradiation and to a different behavior of **(II)**, which exerted cytotoxic effects in the dark. Complex **(I)** has potential application as a photosensitizer, whereas **(II)** seems to behave differently, with no photosensitizing potential. The application of **(I)** in PDT needs to be exploited in other cell lines.

## Data Availability Statement

The original contributions presented in the study are included in the article/Supplementary Material, further inquiries can be directed to the corresponding author/s.

## Author Contributions

TM and LN: designed and performed the experiments. LP: designed and performed the biological experiments. KF: verified and performed the ICP methods. CX and RA: conceived and planned the photophysical experiments. MH, CT, and RS: supervised the findings of this work. All authors discussed the results and contributed to the final manuscript.

## Conflict of Interest

The authors declare that the research was conducted in the absence of any commercial or financial relationships that could be construed as a potential conflict of interest. The reviewer FQ declared a shared affiliation with several of the authors, RS, TM, LN, and LP, to the handling editor at time of review.
