# Supplementary material for: The Influence of Some Axial Ligands on Ruthenium–Phthalocyanine Complexes: Chemical, Photochemical, and Photobiological Properties
Source: Front Mol Biosci. 2021 Jan 12;7:595830. doi: 10.3389/fmolb.2020.595830 (PMC7835839; doi:10.3389/fmolb.2020.595830)
Supplement: Supplementary file 1 [file Data_Sheet_1.pdf]

The influence of some axial ligands on ruthenium-phthalocyanine complexes: chemical, photochemical, and photobiological properties.

Tássia Joi Martins[1,5], Laisa Bonafim Negri[2,3,4], Laena Pernomian[2,6], Kelson do Carmo Freitas Faial[7], Congcong Xue[5], Regina N. Akhimie[5], Michael R. Hamblin[3,4,8*], Claudia Turro[5*] and Roberto S. da Silva[1,2,3,4*]

Supplementary Data

Table 1. Attribution Raman bands for complex **(II)**.

**Raman (cm^{-1})**	**Attribution[1,2]**
1482	ν (–N=)
1414	ν (C–H) isoindol
1349	ν C–N pyrrol
1131	pyrrol ring breathing
1120	ν (C_α–C_β)
954	Ring deformation
730	Pc ring
576	σ out of plane (C–H)
239	Metal–N Pc

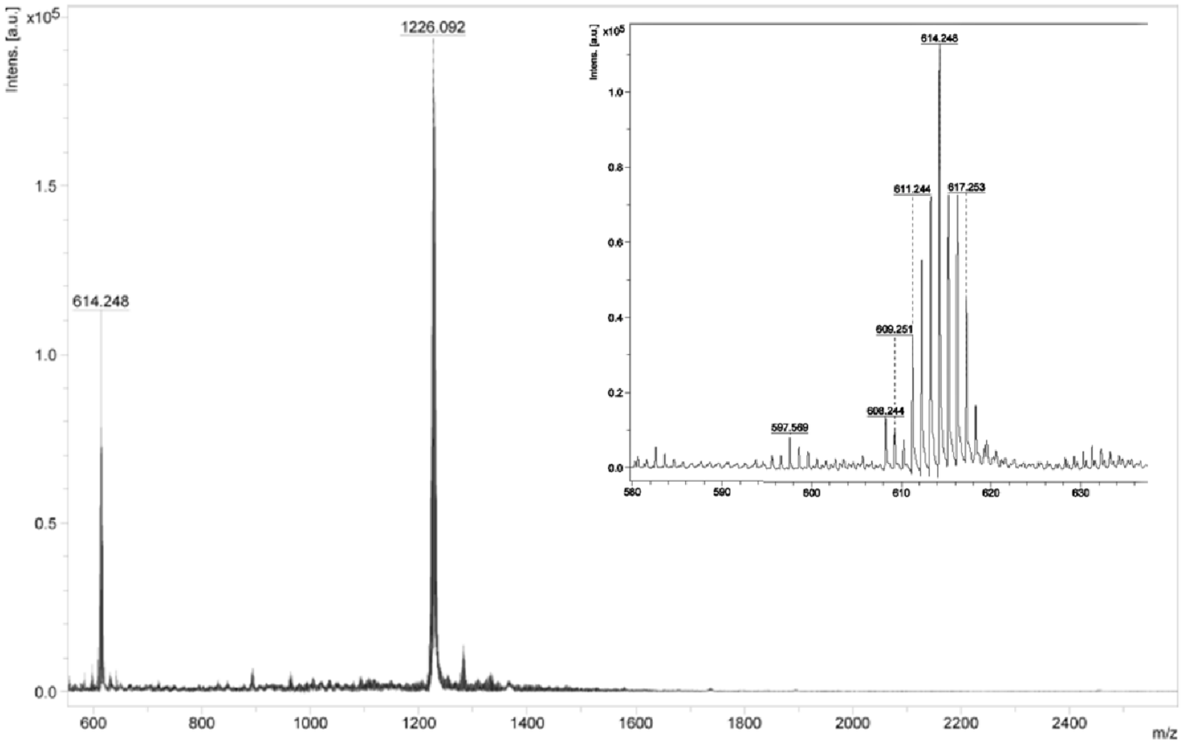

Figure 1. MALDI-TOF spectrum of *trans*-[RuCl(DMSO)(Pc)]. The mass spectrum was obtained by MALDI-TOF / TOF Ultraflextreme (Bruker Daltonics), using 2,5-dihydroxybenzoic acid (DHB) as matrix. It was prepared a solution at concentration of 20 mg/mL in acetonitrile and deionized water containing 0.1% trifluoroacetic acid, with proportion 3:7 v/v. The parameters used were: 100 laser shots per spectrum, *pulsed ion extraction* (PIE) of 100 ns, positive mode, laser frequency of 1000 Hz and negative ionization mode. The applied IS1 and IS2 voltages were 20 kV and 18.05 Kv, respectively. The reflector mode was used with the voltages in RV1 and rv2 being 21.3 Kv and 10.7 Kv. For the external calibration of the equipment, were used a mixture of peptides, from Bruker. Inset: corresponding MS/MS spectrum for m/z 614.248 shows that the dominant fragments arise from [Ru(Pc)].

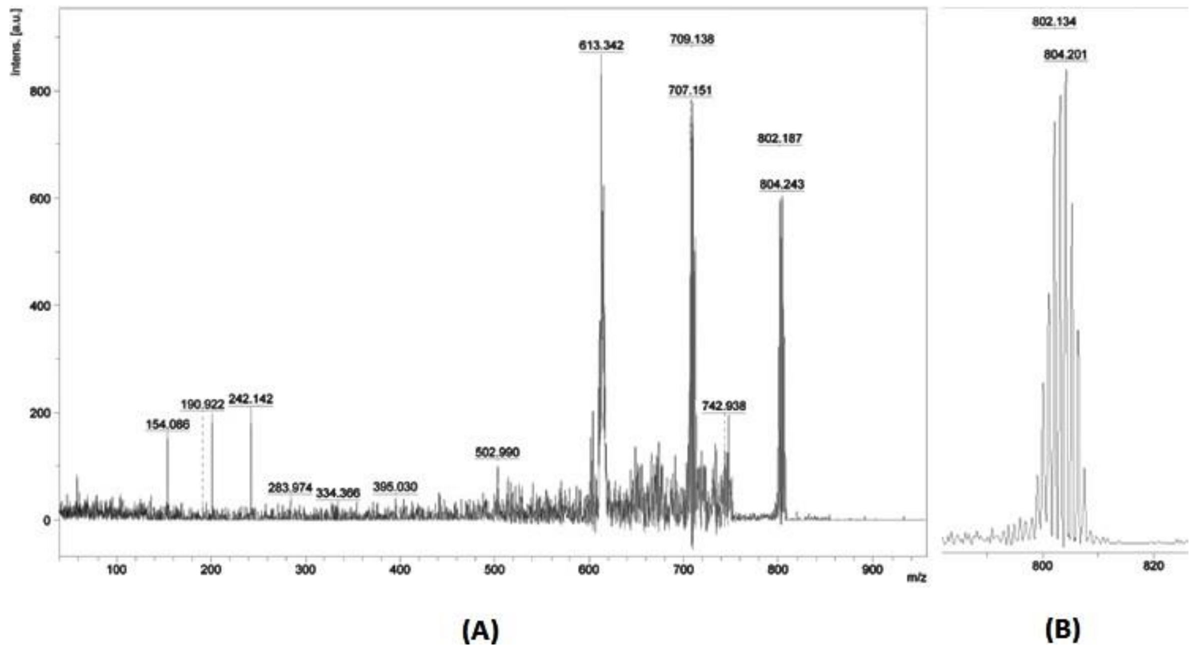

Figure 2. A) MALDI-TOF spectrum of complex *trans*-[Ru(Pc)(4-ampy)$_2$], showing peaks with *m/z* = 614.056 and 802.172. The mass spectrum was obtained by MALDI-TOF / TOF Ultraflextreme (Bruker Daltonics), using 2,5-dihydroxybenzoic acid (DHB) as matrix. It was prepared a solution at concentration of 20 mg/mL in acetonitrile and deionized water containing 0.1% trifluoroacetic acid, with proportion 3:7 v/v. B) corresponding MS/MS spectrum for m/z 802.134 shows that the dominant fragments arise from ruthenium complex fragment. The parameters used were: 100 laser shots per spectrum, *pulsed ion extraction* (PIE) of 100 ns, positive mode, laser frequency of 1000 Hz and negative ionization mode. The applied IS1 and IS2 voltages were 20 kV and 18.05 Kv, respectively. The reflector mode was used with the voltages in RV1 and rv2 being 21.3 Kv and 10.7 Kv. For the external calibration of the equipment, were used a mixture of peptides, from Bruker.

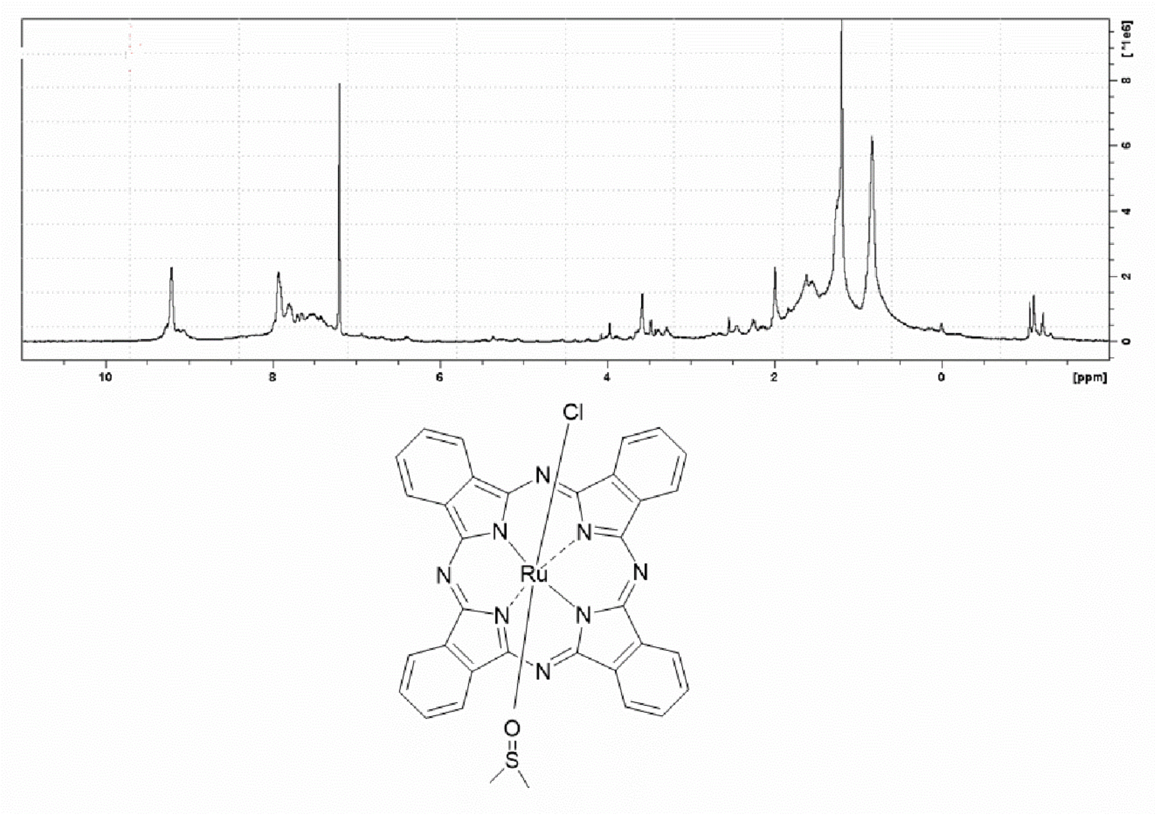

Figure 3. ^{1}H NMR spectrum and attribution signals of *trans*-[RuCl(DMSO)(Pc)]. This spectrum was recorded on a BRUKER@ Avance III 400 to 450 Hz, using $CDCl_3$ as solvent and TMS as internal standard.

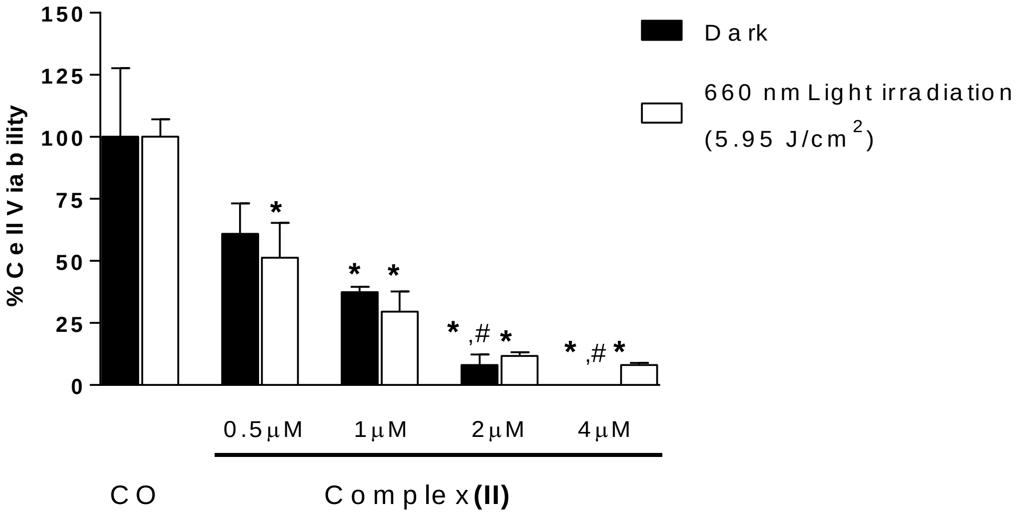

Figure 4. Cell viability of B16F10 treated with complex **(II)**. Cells were incubated with complex **(II)** in different concentrations (0.5 µmol/L to 4 µmol/L) for 24 h and after the treatment, they were irradiated with 660 nm light at 5.95 J/cm^2. CO means control (1% DMSO). Data are presented in percentage (%) of the mean ± S.E.M. values. Two-way ANOVA, with Bonferroni post-hoc ($P<0.05$). * different from respective control (CO); # different from complex **(II)** at 0.5 µM in the dark. All the results in this figure are representative of independent experiments (n=3-4).

Table 2. Intracellular measurement of ruthenium in B16F10 cells treated with complex **(II)**, by ICP-OES.

**B16F10 cells: intracellular ruthenium (ng/µL)**	
Complex **(II)**: 1 µmol/L, 24h	0.8584
Control (1% DMSO), 24h	*n.d.*

n.d. means not detected.

It was analyzed by ICP-OES technique 50 µL of B16F10 cell extract, in culture medium, treated with complex **(II)** or vehicle (control condition; 1% dimethylsulfoxide – DMSO) for 24h. Ruthenium standard suitable for spectrophotometric analysis AA (AARU1000V, Specsol) was used to standard curve. All the samples were diluted in milliQ water.

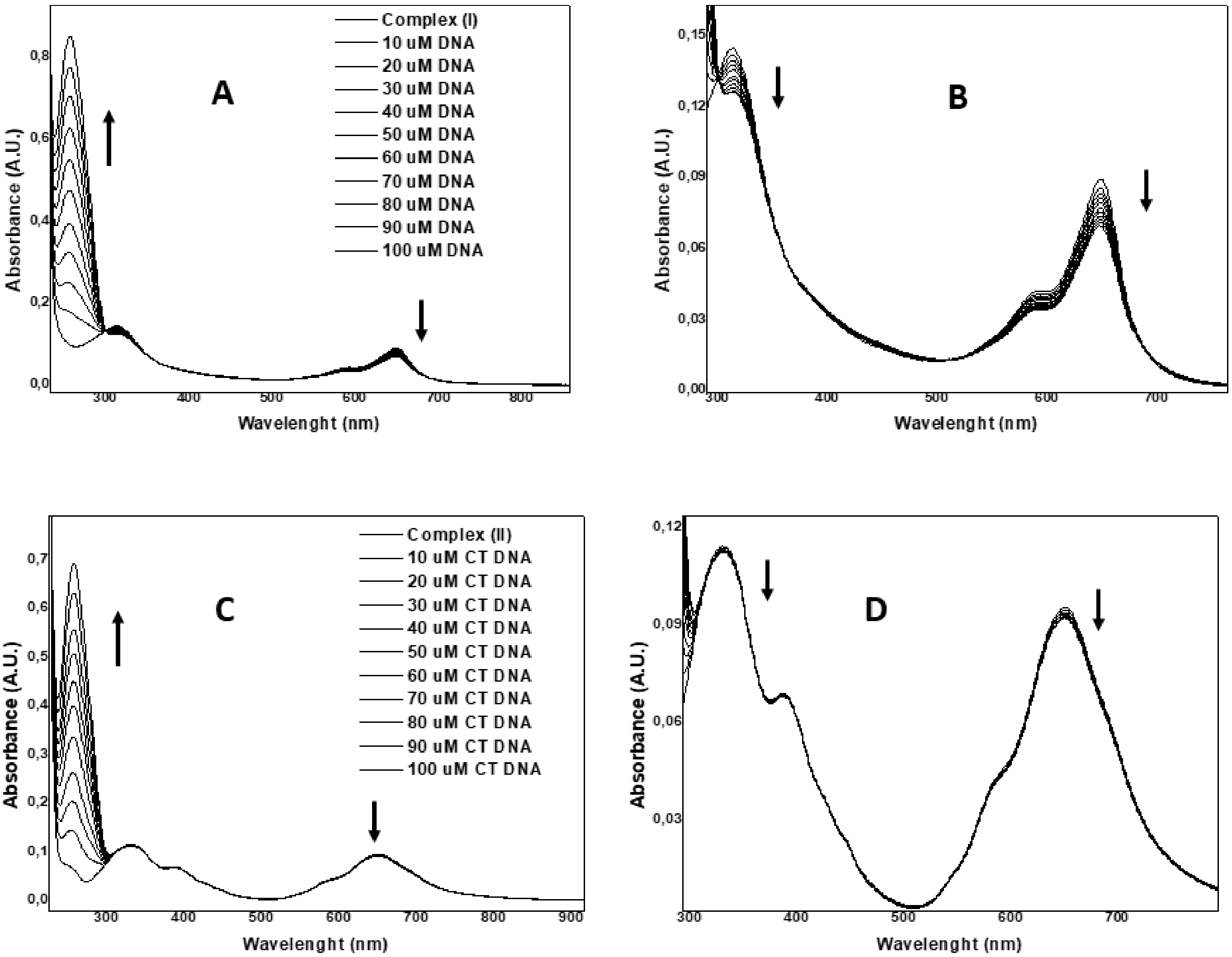

Figure 5. Spectrophotometric titration for complexes **(I)** and **(II)** and calf thymus (CT) DNA. In figures **A** and **C** it is possible to observe the band at 260 nm referring to calf thymus (CT) DNA. Figures **B** and **D** present the hypochromic effect of the bands related to the complexes **(I)** and **(II)**, respectively.

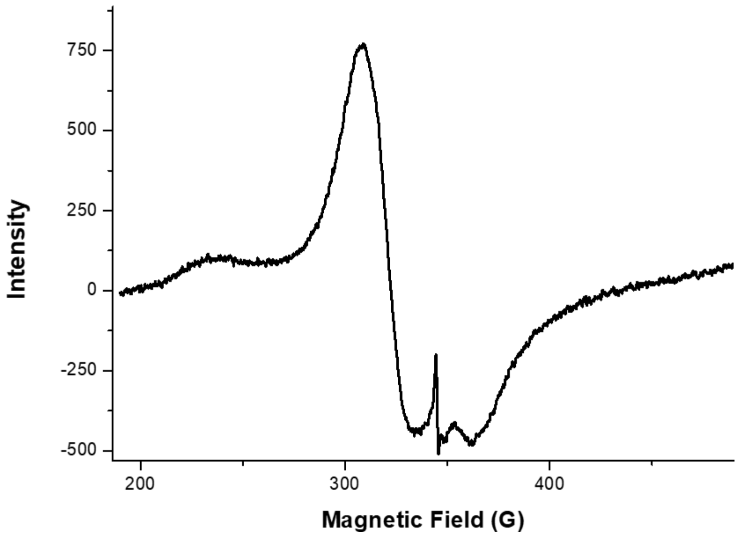

Figure 6. Electronic Paramagnetic Resonance **(**EPR) spectrum of *trans*-[RuCl(DMSO)(Pc)]. EPR measurements were carried out on solution using a capillary glass tube, using the equipment JEOL JESFA200 spectrometer in an X-Band (9,4 GHz) with a scanning field of 10 G, scan time of 4 minutes, modulation amplitude of 100 kHz, modulation frequency of 100 kHz and microwave power of 10 mW.

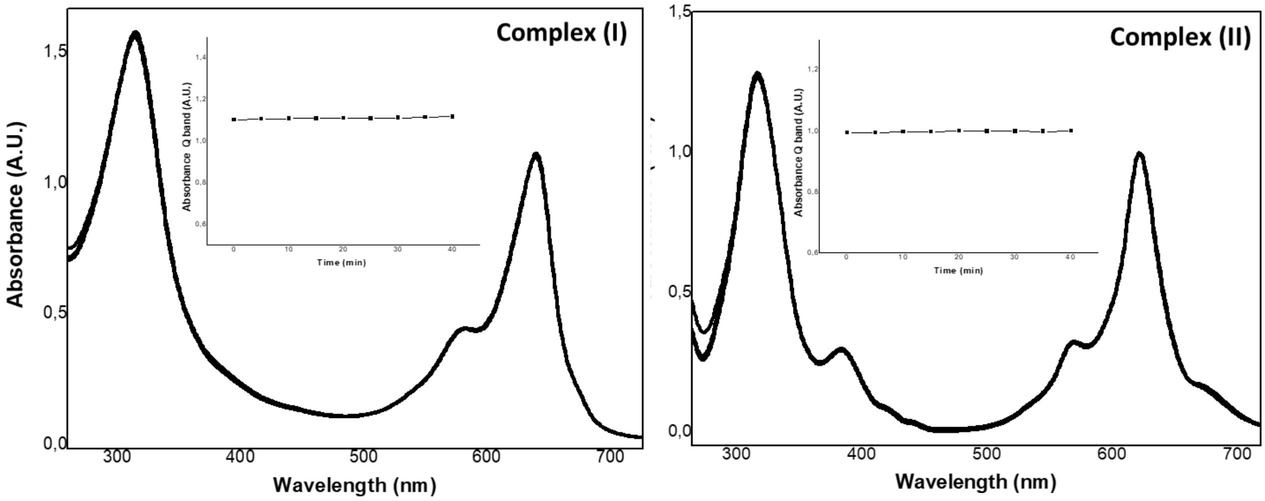

Figure 7. Electronic absorption spectra of complexes **(I)** and **(II)**, respectively, after continuously irradiation for 40 minutes.

REFERENCES

1. SAINI, G. S. S.; SHARMA, S.; KAUR, S.; TRIPATHI, S. K.; MAHAJAN, C. G.; Infrared spectroscopic studies of free-basetetraphenylporphine and its dication, **Spectrochimica Acta Part A** 61, *2004*, 3070-3076.

2. LI, X.; XU, W.;WANG, X.; JIA, H.; ZHAO, B.; LI, B.; OZAKI, Y.; Ultraviolet-visible and surface-enhanced Raman scattering spectroscopy studies on self-assembled films of ruthenium phthalocyanine on organic monolayer-modified silver substrates, **Thin Solid Films**, 457, 372–380, 2004.
